# Gold Nanoparticles in Neurological Diseases: A Review of Neuroprotection

**DOI:** 10.3390/ijms25042360

**Published:** 2024-02-17

**Authors:** Ming-Chang Chiang, Yu-Ping Yang, Christopher J. B. Nicol, Chieh-Ju Wang

**Affiliations:** 1 Department of Life Science, College of Science and Engineering, Fu Jen Catholic University, New Taipei City 242, Taiwan; 2 Sylvester Comprehensive Cancer Center, Miller School of Medicine, University of Miami, Miami, FL 33136, USA; yyang22@med.miami.edu; 3 Department of Biochemistry and Molecular Biology, Miller School of Medicine, University of Miami, Miami, FL 33136, USA; 4 Departments of Pathology & Molecular Medicine and Biomedical & Molecular Sciences, Cancer Biology and Genetics Division, Cancer Research Institute, Queen’s University, Kingston, ON K7L 3N6, Canada; nicolc@queensu.ca

**Keywords:** gold nanoparticles, neuroprotective effects, anti-neuro-inflammatory toxicity, Alzheimer’s disease, Parkinson’s disease and stroke

## Abstract

This review explores the diverse applications of gold nanoparticles (AuNPs) in neurological diseases, with a specific focus on Alzheimer’s disease (AD), Parkinson’s disease (PD), and stroke. The introduction highlights the pivotal role of neuroinflammation in these disorders and introduces the unique properties of AuNPs. The review’s core examines the mechanisms by which AuNPs exert neuroprotection and anti-neuro-inflammatory effects, elucidating various pathways through which they manifest these properties. The potential therapeutic applications of AuNPs in AD are discussed, shedding light on promising avenues for therapy. This review also explores the prospects of utilizing AuNPs in PD interventions, presenting a hopeful outlook for future treatments. Additionally, the review delves into the potential of AuNPs in providing neuroprotection after strokes, emphasizing their significance in mitigating cerebrovascular accidents’ aftermath. Experimental findings from cellular and animal models are consolidated to provide a comprehensive overview of AuNPs’ effectiveness, offering insights into their impact at both the cellular and in vivo levels. This review enhances our understanding of AuNPs’ applications in neurological diseases and lays the groundwork for innovative therapeutic strategies in neurology.

## 1. Introduction

### 1.1. The Role of Neuroinflammation in Neurodegenerative Diseases and Stroke

Neurological disorders encompass a diverse range of conditions that affect the intricate network of the nervous system, leading to disruptions in cognitive, motor, sensory, and autonomic functions [1,2]. Within this complex landscape, neuroinflammation, toxicity, and the quest for neuroprotection have emerged as central themes that greatly influence disease progression and therapeutic strategies [3]. Neuroinflammation, characterized by the activation of immune responses within the central nervous system, plays a pivotal role in the initiation and progression of various neurological disorders [4]. Inflammatory processes involving microglia, astrocytes, and immune cells can release pro-inflammatory cytokines, chemokines, and reactive oxygen species, which contribute to neuronal damage, synaptic dysfunction, and ultimately, disease manifestation [5,6,7,8]. The intricate interplay between immune cells and neurons highlights the need to decipher the underlying neuroinflammation mechanisms to develop targeted interventions [9]. In neurodegenerative diseases like AD, PD, and stroke, neuroinflammation can contribute to the progression of damage [10,11,12]. AuNPs have attracted attention in biomedical research due to their unique properties, including their small size, large surface area, and tunable surface chemistry [13]. These properties make them suitable for various applications, including drug delivery, imaging, and therapy. In this comprehensive review, the multifaceted applications of AuNPs in neurological diseases are explored, with a particular focus on AD, PD, and stroke. The core of this review is to provide insights into the complex mechanisms by which AuNPs exert neuroprotective and anti-neuro-inflammatory effects. Aili et al. (2023) conducted a study that demonstrated the potential of AuNPs to reduce neuroinflammation and inhibit oxidative stress in AD [14]. The potential neuroprotective effect of AuNPs against PD is demonstrated by their ability to decrease inflammation in in vitro and in vivo models [15]. Additionally, Salatin et al. found that AuNPs have a neuroprotective effect in ischemic stroke models [16]. These findings highlight the potential of AuNPs as a promising avenue for future neurological treatments.

### 1.2. Properties and Applications of AuNPs for Biomedical Applications

AuNPs possess unique properties that make them highly suitable for various biomedical applications [17,18,19]. These properties stem from their nanoscale size, tunable surface chemistry, and distinctive optical and electronic characteristics [20,21]. AuNPs display distinctive characteristics owing to their minute size and exceptional structure. They have a small size and high surface area: AuNPs typically have diameters ranging from 1 to 100 nanometers, allowing them to interact with biomolecules at the cellular and molecular levels. Their high surface-area-to-volume ratio enables efficient drug loading and interaction with target molecules [22]. The size, shape, and surface modification of AuNPs influence their navigation in the bloodstream, interaction with immune cells, and enhanced permeability and retention [22]. The small size of AuNPs enables them to penetrate biological barriers, including cell membranes [23]. This size range is ideal for interactions at the cellular and molecular levels. Due to their small size, AuNPs are easily internalized by cells through processes such as endocytosis [24]. This cellular uptake facilitates the targeted delivery of therapeutic or imaging agents directly into cells. The small size of AuNPs enables them to interact with biomolecules such as proteins, nucleic acids, and lipids [25,26]. These interactions can be used in various applications, including drug delivery, imaging, and diagnostics. The high surface-area-to-volume ratio of AuNPs is a critical factor in their reactivity and functionality [27]. This property allows many functional groups to be attached to the surface of the nanoparticles, enhancing their ability to bind and interact with target molecules. The large surface area provides ample space for the attachment of therapeutic agents, making AuNPs effective carriers for drug delivery. This property is particularly advantageous in nanomedicine, which often requires precise and controlled drug release. The high surface area of AuNPs contributes to their catalytic activity [28]. This property has been explored in various applications, including catalysis of chemical transformations and platforms for enhanced bioanalysis. In summary, the small size and high surface area of AuNPs make them versatile nanomaterials that are capable of interacting with biological systems at different scales [29]. These properties underpin their applications in drug delivery, imaging, diagnostics, and other biomedical fields.

Additional factors that make AuNPs advantageous for biomedical applications include Biocompatibility: gold is considered biocompatible and relatively inert, minimizing the potential for adverse immune responses or cytotoxicity when used in biological systems [30]. Surface modification: The surface of AuNPs can be easily functionalized with various molecules, such as targeting ligands, antibodies, or drugs [31]. This ability allows researchers to customize AuNPs for specific applications, thus enhancing their targeting and therapeutic capabilities. Drug delivery: AuNPs can encapsulate therapeutic compounds, protecting them from degradation and enhancing their delivery to target cells or tissues [32]. The surface modification of AuNPs allows for drug-controlled release, improving their bioavailability and efficacy. Crossing biological barriers: by functionalizing their surfaces, AuNPs can be engineered to cross the blood–brain barrier (BBB) and target specific cells or tissues, expanding their potential for neurology and other applications [33]. These unique properties make AuNPs versatile tools for various biomedical applications [34].

The toxicity and safety profile of AuNPs is a critical aspect that requires thorough investigation, especially in long-term neurological applications. While AuNPs hold promise for various therapeutic purposes, potential adverse effects must be carefully considered. The size of AuNPs has been identified as a crucial factor influencing their toxicity [30]. Smaller nanoparticles may exhibit higher toxicity due to increased surface reactivity and potential to induce cellular stress. Understanding the size-dependent toxicity is essential for designing safe AuNP formulations. The biodistribution and clearance of AuNPs from the body can impact their safety. Small nanoparticles might undergo rapid renal clearance, affecting their systemic circulation and potential accumulation in vital organs, including the brain. A comprehensive understanding of AuNPs’ pharmacokinetics is crucial for assessing their long-term safety [35]. Surface modifications with biocompatible coatings aim to enhance the safety profile of AuNPs. Coating AuNPs with materials such as polymers or surfactants improves their biocompatibility and helps mitigate potential toxic effects that are associated with the bare surface of nanoparticles [36]. Assessing the direct impact of AuNPs on neuronal cells is crucial for evaluating their neurotoxicity. Studies should investigate potential disruptions of neuronal function, cellular viability, and synaptic activity. Additionally, understanding the long-term effects of AuNPs on neural networks is essential for predicting their safety in neurological applications. AuNPs can potentially elicit immunological responses, leading to inflammation or immune activation. Chronic inflammation is particularly concerning in the context of neurological diseases. Investigating the immunomodulatory effects of AuNPs is crucial for predicting their long-term impact on the nervous system [37]. Given the potential use of AuNPs for drug delivery to the brain, maintaining the integrity of the BBB is critical. Assessing the impact of AuNPs on the BBB’s permeability and function over an extended period is essential to ensure the safety of long-term applications. Studies examining the genotoxic and mutagenic potential of AuNPs are necessary for evaluating their safety. Conducting long-term in vivo studies in animal models is essential for assessing the chronic effects of AuNPs on the nervous system. Preclinical studies should be designed to mimic real-world scenarios and assess the safety of AuNPs over extended periods to ensure their suitability for long-term neurological interventions. In conclusion, the safety profile of AuNPs in long-term neurological applications is a complex consideration involving size, surface modifications, immunological responses, and potential neurotoxicity. Rigorous and comprehensive studies, especially those conducted over extended periods, are imperative to advance our understanding of the safety aspects of AuNPs and pave the way for their responsible and effective use in neurological therapeutics.

Comparing the efficacy and safety of AuNPs with other nanomaterials that are used in neurology provides insights into their relative advantages and limitations. Several nanomaterials have been explored for neurological applications, each with unique properties. Here, we compare AuNPs with commonly studied nanomaterials, including carbon-based nanomaterials and polymeric nanoparticles [38,39]. AuNPs vs. carbon-based nanomaterials: Advantages of AuNPs: AuNPs are versatile and quickly functionalized, allowing for tailored surface modifications. The optical properties of AuNPs make them suitable for imaging applications. Surface modifications can enhance their biocompatibility and reduce toxicity. Limitations of AuNPs: AuNPs have limited drug-loading capacity compared to some carbon-based nanomaterials. They may have lower electrical conductivity compared to carbon-based nanomaterials. Advantages of carbon-based nanomaterials: Carbon-based nanomaterials have a high surface area and excellent drug-loading capacity [40]. They have superior electrical conductivity for neural interfacing applications and potential for multifunctionality in drug delivery and imaging. Limitations of carbon-based nanomaterials: there are more significant concerns about their biocompatibility and toxicity, controlling their size, and dispersibility challenges regarding these materials [41]. AuNPs vs. polymeric nanoparticles: Advantages of AuNPs: AuNPs have unique optical properties for imaging and potential for targeted drug delivery. Surface modifications for enhanced biocompatibility are possible. Limitations of AuNPs: Polymeric nanoparticles may offer higher drug-loading capacities. There is limited control over AuNPs release kinetics compared to some polymeric systems. Advantages of polymeric nanoparticles: We have excellent control over the drug release kinetics of polymeric nanoparticles. They are versatile in terms of structure and composition [42] and can be designed to provide sustained release profiles. Limitations of polymeric nanoparticles: There are challenges in terms of achieving precise targeting. They may have lower stability compared to AuNPs. Overall considerations: Efficacy: The choice of nanomaterial depends on the specific application and the therapeutic payload. AuNPs excel in imaging applications and targeted drug delivery, while other nanomaterials may offer advantages in drug loading or release kinetics. Safety: Surface modifications significantly impact the safety profile. AuNPs with biocompatible coatings may have advantages in terms of reduced toxicity. Carbon-based nanomaterials may raise concerns about their long-term biocompatibility, while polymeric nanoparticles are generally considered safe with appropriate formulations. AuNPs, due to their surface modifications and versatile properties, may have advantages in terms of customization for clinical applications. In conclusion, the choice between AuNPs and other nanomaterials depends on the specific requirements of the neurological application [43]. While AuNPs offer unique advantages, such as optical properties and ease of functionalization, the selection should be based on careful consideration of the desired therapeutic outcomes and safety considerations in neurology.

### 1.3. Effect of Size and Charge of AuNPs or Surface Modification of AuNPs in Neuroprotection

AuNPs have attracted considerable attention across diverse biomedical applications, such as neuroprotection. The size, charge, and surface modification of AuNPs are pivotal determinants of their efficacy in neuroprotection [44]. The initial issue concerns the size of AuNPs regarding penetration and cellular uptake. Small AuNPs show potential in penetrating biological barriers, like the BBB, to reach target neurons more efficiently [45]. Larger nanoparticles may have restricted penetration but could provide longer circulation times within the bloodstream [46]. Interaction with biomolecules: Small-sized AuNPs may have a larger surface area for interacting with biomolecules, potentially influencing their bioavailability and cellular interactions [47]. The surface plasmon resonance (SPR) is impacted by the size of AuNPs, which can affect interactions with light and other molecules [48]. This property could have implications for therapies involving light-based interventions.

Another factor is the surface modification of AuNPs using polyethylene glycol (PEG) coating. PEGylation can improve the stability and biocompatibility of AuNPs [49]. PEG-coated AuNPs can have prolonged circulation times in the bloodstream. Biomaterial coating: Coating AuNPs with biomaterials such as proteins or peptides can increase their specificity for specific cell types or tissues [30]. This can reduce potential immune responses and improve their biocompatibility. Targeted ligands: functionalizing the surface of AuNPs with ligands that target specific receptors on neuronal cells can increase their specificity and efficacy in neuroprotection [50,51]. The most important mechanisms are the neuroprotective mechanisms of AuNPs, anti-inflammatory effects. AuNPs may modulate inflammatory responses, potentially reducing neuroinflammation [52,53]. Antioxidant properties: AuNPs may have inherent antioxidant properties, which may be beneficial in protecting neurons from oxidative stress [54,55]. Drug delivery: surface modifications may enable AuNPs to carry and deliver neuroprotective drugs to specific sites in the brain [56]. Photothermal therapy: small-sized AuNPs with specific surface modifications can be used for photothermal therapy, where light generates localized heat for therapeutic purposes [57]. It is important to note that while AuNPs hold promise for neuroprotection, thorough in vitro and in vivo studies, considering various parameters, are needed to understand their safety and efficacy in specific applications. The field of nanomedicine is rapidly evolving, and ongoing research continues to shed light on the intricate interactions between nanoparticles and biological systems.

The impact of nanoparticles’ size is a critical aspect of the design and application of nanoparticles in neurological research. The size of nanoparticles, including AuNPs, is crucial for their efficacy and safety in neurological applications [58]. The size of nanoparticles significantly influences their ability to penetrate the BBB, a critical factor in delivering therapeutic agents to the central nervous system. Small-sized nanoparticles are more likely to cross the BBB than larger counterparts, enabling targeted drug delivery to the brain [59]. Nanoparticles’ size affects cellular uptake and distribution within the nervous system. Small nanoparticles can efficiently enter cells and interact with intracellular components, potentially influencing cellular processes that are relevant to neuroprotection and anti-neuroinflammation [60]. The size of nanoparticles may impact their anti-neuro-inflammatory effects. Small nanoparticles can potentially interact with microglia and astrocytes more effectively, modulating inflammatory responses. Understanding the optimal size range for anti-neuro-inflammatory effects is crucial for therapeutic design. The size of nanoparticles also affects the amount of therapeutic payload that they can carry. While smaller nanoparticles may have limitations in their payload capacity, their ability to penetrate tissues and cells could compensate for this. Balancing size considerations with payload loading is essential for achieving optimal therapeutic outcomes. The size of nanoparticles influences their biodistribution and clearance from the body. Smaller nanoparticles may be more prone to rapid renal clearance, affecting their systemic circulation and, consequently, their availability in the target tissues [61]. Consideration of the size of biodistribution is crucial for sustained therapeutic effects. The size of nanoparticles dictates their surface area and, consequently, their interactions with biological molecules. Small nanoparticles may have a higher surface area per unit volume, potentially leading to increased interactions with proteins, enzymes, and other biomolecules. This can influence the nanoparticles’ stability, biocompatibility, and safety profile. Size-related toxicity is a significant consideration in nanoparticle research [62]. Small nanoparticles may exhibit different biological behaviors and interactions than larger ones, potentially leading to unexpected toxic effects. Rigorous safety assessments, including long-term studies, are crucial to ensuring the safe use of nanoparticles in neurological applications. The size of AuNPs can influence their optical and magnetic properties. Small AuNPs may exhibit unique characteristics that are advantageous for imaging and therapeutic purposes. Understanding size-dependent properties contributes to the development of multifunctional nanoparticles for neurological applications. In conclusion, nanoparticles’ size is a critical determinant of their efficacy and safety in neurological applications.

Surface modifications of AuNPs play a pivotal role in determining their interaction with neurological targets, influencing their therapeutic potential. These modifications involve altering the surface chemistry of AuNPs through the attachment of various functional groups or biomolecules [63]. Surface modifications with biocompatible materials, such as polymers or surfactants, improve the overall biocompatibility of AuNPs [64]. This is crucial for minimizing adverse effects and ensuring compatibility with the intricate environment of the nervous system. Certain surface modifications can enhance the ability of AuNPs to traverse the BBB. Coating AuNPs with molecules that facilitate receptor-mediated transcytosis or exploit endogenous transport mechanisms can improve their penetration into the brain, allowing for targeted drug delivery [65]. Functionalizing AuNPs with ligands or peptides that have an affinity for specific receptors on neuronal cells enables targeted interactions. This approach enhances the specificity of AuNPs for particular cell types within the nervous system, optimizing therapeutic effects while minimizing off-target interactions. Surface modifications influence the cellular uptake of AuNPs. The type and density of functional groups on the surface can alter the interactions with cell membrane components, affecting internalization pathways. This can be tailored for efficient uptake by specific cell types within the nervous system. Surface modifications can impart anti-inflammatory properties to AuNPs. Coating with anti-inflammatory agents, such as specific peptides or drugs, enhances the ability of AuNPs to modulate neuro-inflammatory responses, offering a targeted therapeutic approach for neurodegenerative diseases. Surface modifications impact the intracellular fate of AuNPs. For instance, modifying the surface with materials that facilitate endosomal escape can enhance the delivery of therapeutic payloads to the cytoplasm, improving the efficacy of AuNPs in influencing cellular processes [26]. Surface modifications allow for the integration of imaging agents or therapeutic molecules onto AuNPs, creating multifunctional nanoparticles. Coating AuNPs with biocompatible materials helps mitigate potential immunogenic responses [32]. This is essential for the long-term application of AuNPs in neurological therapies, reducing the risk of adverse reactions or immune-related side effects. Surface modifications contribute to the stability of AuNPs in physiological conditions, preventing aggregation and ensuring their long circulation time in the bloodstream. This is crucial for sustained delivery to target sites within the nervous system [66]. In summary, surface modifications of AuNPs are intricately linked to their interaction with neurological targets and significantly influence their therapeutic potential. Customizing surface properties opens avenues for developing AuNPs with enhanced specificity, improved biocompatibility, and multifunctionality, contributing to advancing targeted and effective therapies for neurological diseases. Ongoing research in this area continues to uncover novel surface modification strategies that optimize AuNPs for neurological applications.

## 2. Mechanisms of Neuroprotection and Anti-Neuro-Inflammatory Effects of AuNPs

**Figure 1:**
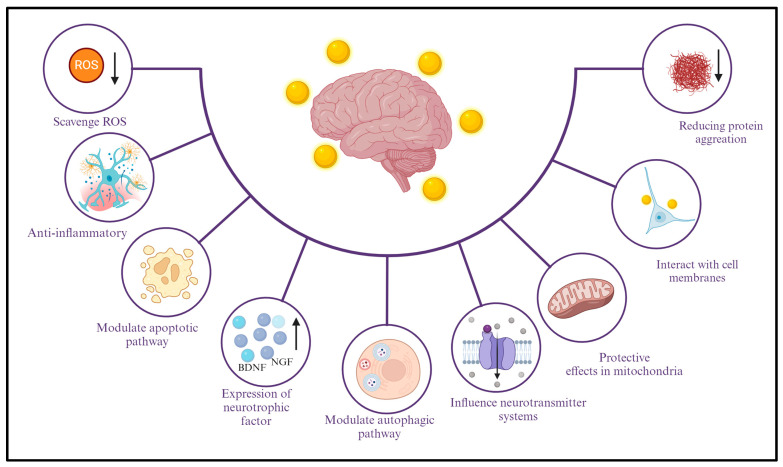
The neuroprotective effects of AuNPs involve intricate interactions at the cellular and molecular levels, with ongoing research proposing various mechanisms. These mechanisms vary based on AuNPs’ size, shape, and surface modifications and the specific neurological disorder context. Continued research emphasizes the antioxidant, anti-inflammatory, modulate apoptotic pathway and protective effects in mitochondria of AuNPs, supporting their therapeutic potential in neurodegenerative diseases. “[Up Arrow image], increase; [Down Arrow image], decrease.” Figure 1 was created with BioRender.

The molecular mechanisms underlying the neuroprotective effects of AuNPs are multifaceted and involve intricate interactions at the cellular and molecular levels (Figure 1). While research in this area is ongoing, several potential mechanisms have been proposed based on preclinical studies. Below are some of the molecular mechanisms that are associated with the neuroprotective effects of AuNPs. It has been suggested that AuNPs possess inherent antioxidant properties, which could contribute to their neuroprotective effects [67]. They may scavenge reactive oxygen species (ROS) and reduce oxidative stress, a critical factor in the pathogenesis of neurodegenerative diseases [53]. AuNPs may exert anti-inflammatory effects by modulating various signaling pathways [14]. They could interfere with the activation of microglia and astrocytes, reducing the release of pro-inflammatory cytokines such as tumor necrosis factor-alpha (TNF-α) and interleukin-1 beta (IL-1β). This modulation of neuroinflammation is crucial for neuroprotection. AuNPs have demonstrated the ability to modulate apoptotic pathways, contributing to neuroprotection [67]. They may influence critical regulators of apoptosis, such as Bcl-2 family proteins and caspases, thereby promoting cell survival and preventing neuronal apoptosis in neurodegenerative conditions [68]. Mitochondrial dysfunction is implicated in various neurological disorders. AuNPs may exert protective effects on mitochondria, maintaining their function [68] and preventing the release of damaging reactive species. This contributes to overall cellular homeostasis. AuNPs may interact with cell membranes, influencing membrane fluidity and stability. This interaction could have downstream effects on intracellular signaling pathways, leading to neuroprotective responses. AuNPs may interfere with the aggregation of misfolded proteins, such as amyloid-beta (Aβ) in AD or alpha-synuclein in PD [66].

By preventing or reducing protein aggregation, AuNPs contribute to the preservation of the neuronal structure and function. It is important to note that the specific molecular mechanisms may vary depending on factors such as the AuNPs’ size, shape, and surface modifications and the context of the neurological disorder that is under investigation. Continued research is essential to unravel these mechanisms’ intricate details and validate the neuroprotective potential of AuNPs for therapeutic applications, i.e., to gain evidence for the efficacy of AuNPs in neurology. In studies on neurological conditions in animal models, AuNPs exhibit superior neuroprotective mechanisms. With antioxidant properties, anti-inflammatory mechanisms, and interactions with specific cellular pathways, this could help build a mechanistic understanding of the efficacy of AuNPs in neurology. The da Silva et al. (2024) study explores the potential neuroprotective effects of AuNPs (70 µg/kg) reducing oxidative stress in the hippocampus in an obesity-induced animal model [69]. Silveira et al. (2023) explore the highlighted anti-inflammatory and antioxidant effects of AuNPs, suggesting their potential as a therapeutic agent in treating neurodegenerative diseases [70].

### 2.1. Explanation of the Different Mechanisms through Which AuNPs Exhibit Neuroprotective Effects

AuNPs have shown promising neuroprotective effects through several distinct mechanisms, each contributing to preserving and enhancing neuronal viability and function [67]. Some of the fundamental mechanisms through which AuNPs exert their neuroprotective effects [15] are presented below. By dampening excessive inflammation, AuNPs help prevent further neuronal damage and reduce neuroinflammation-associated neurotoxicity [53]. Antioxidant properties: AuNPs possess inherent antioxidant capabilities due to their ability to scavenge ROS, thereby preventing oxidative stress [71]. Oxidative stress is a significant contributor to neurodegenerative disorders, and by neutralizing ROS, AuNPs help mitigate oxidative damage to neurons and maintain cellular homeostasis [55]. Mitochondrial dysfunction in the context of neurodegenerative diseases: Mitochondrial dysfunction is prevalent in neurodegenerative diseases. AuNPs have been shown to stabilize mitochondrial membrane potential, improve mitochondrial respiration, and enhance ATP production [68]. These effects contribute to the maintenance of cellular energy and neuronal health. Antiapoptotic effects: AuNPs can inhibit apoptotic pathways, preventing the programmed cell death of neurons [68]. By modulating the expression of apoptotic proteins and suppressing caspase activation, AuNPs promote neuronal survival and inhibit cell death cascades [72]. It should be noted that while there is evidence supporting the potential of gold nanoparticles in promoting apoptosis for cancer therapeutic purposes [73,74], this area is still under active research. Therefore, the use of such nanoparticles in clinical settings requires further study.

Neurotrophic effects: AuNPs can enhance the secretion of neurotrophic factors, such as brain-derived neurotrophic factor (BDNF) and nerve growth factor (NGF), which promote neuronal growth, survival, and differentiation [75]. These factors are crucial for maintaining neuronal health and plasticity. Enhanced neuronal connectivity: AuNPs have been shown to promote neurite outgrowth and enhance synaptic connectivity between neurons [76]. This effect is essential for maintaining neural circuits and facilitating communication between neurons. Modulating signaling pathways: AuNPs can modulate intracellular signaling pathways that are involved in cell survival and neuroprotection. By influencing pathways such as PI3K/Akt and MAPK, AuNPs promote prosurvival signals and inhibit proapoptotic signals [77]. Reduction in protein aggregation: AuNPs have been investigated for their potential to reduce the aggregation of misfolded proteins, which are implicated in many neurodegenerative diseases. By preventing protein aggregation, AuNPs help mitigate the toxic effects of protein aggregates on neurons [66]. Enhanced BBB permeability: Functionalized AuNPs can cross the BBB and deliver therapeutic payloads to the CNS [78,79]. This allows for targeted treatment of neurodegenerative disorders directly at the site of action. Photothermal effects: In photothermal therapy, AuNPs can be selectively heated using near-infrared light, leading to localized hyperthermia [73]. This can trigger the release of heat shock proteins, which have neuroprotective properties.

Together, these mechanisms contribute to the neuroprotective effects of AuNPs, making them promising candidates for therapeutic intervention in neurological diseases [71,80,81]. However, the specific tools may vary depending on the properties of AuNPs, such as their size, shape, surface functionalization, and neural environment context. While these mechanisms show promise, further research is needed to fully understand how AuNPs interact with the nervous system and optimize their potential neuroprotective therapeutic applications.

### 2.2. Anti-Inflammatory Properties of AuNPs

AuNPs have recently received widespread attention as multifunctional nanomaterials and have broad application prospects in neuroscience [67,82]. Their unique physicochemical properties, tunable surface chemistry, and biocompatibility make them attractive candidates for addressing the complex challenges of neuroinflammation and neuroprotection in various neurological diseases [33,83]. Additionally, AuNPs show promising anti-inflammatory properties, mainly due to their ability to modulate immune responses and interact with multiple cellular pathways [84]. Cytokine regulation: AuNPs can regulate the production and release of cytokine signaling molecules that are involved in inflammation. The inhibition of pro-inflammatory cytokine expression (e.g., TNF-α, IL-1β) and the promotion of anti-inflammatory cytokines (e.g., IL-10) may co-occur [14,53]. This helps maintain a balanced immune response. Microglial activation: Microglia are immune cells in the brain that are responsible for neuroinflammation. AuNPs can modulate microglial activation, leading to decreased release of inflammatory mediators. NF-κB pathway inhibition: AuNPs can prevent activation of the NF-κB signaling pathway, a critical regulator of inflammation [85]. By blocking NF-κB activation, AuNPs reduce the expression of inflammatory genes [53]. MAPK signaling modulation: Mitogen-activated protein kinases (MAPKs) are critical mediators of inflammatory responses. AuNPs can affect MAPK phosphorylation, altering the pro- and anti-inflammatory signaling balance. This modulation of MAPKs contributes to the overall anti-inflammatory effect of AuNPs. AuNPs may downregulate JAK/STAT signaling, reducing the expression of inflammatory cytokines and promoting an anti-inflammatory milieu. Toll-like receptor (TLR) modulation: TLRs initiate immune responses upon pathogen recognition. AuNPs have been shown to interfere with TLR activation, inhibiting downstream inflammatory signaling. This modulation of TLRs directly influences the initiation of neuro-inflammatory cascades. Inhibition of inflammasome activation: AuNPs have been shown to inhibit the assembly and activation of inflammasomes, multi-protein complexes that trigger the release of pro-inflammatory cytokines. Cellular uptake and signaling: AuNPs can enter cells and interact with signaling pathways that are involved in inflammation, influencing cellular responses and gene expression [14,37,53]. These references suggest that AuNPs exhibit anti-inflammatory properties, making them promising candidates for therapeutic intervention against diseases that are characterized by inflammation, such as AD and autoimmune diseases. The multifaceted roles of gold nanoparticles in regulating inflammation and oxidative stress highlight their potential for biomedical applications. The interplay of these pathways and signaling mechanisms underscores the multifaceted nature of AuNPs’ anti-neuro-inflammatory effects. The precise mechanisms may vary depending on nanoparticle properties, cell type, and the specific context of neuroinflammation. Understanding these mechanisms is essential for harnessing the full potential of AuNPs as therapeutic agents to combat neuroinflammation in various neurological disorders.

## 3. Application of AuNPs in AD, PD, and Stroke

The potential applications of AuNPs in neurological disorders such as AD, PD, and stroke [86,87,88] will be discussed in this section. AuNPs have attracted attention in nanomedicine due to their unique physicochemical properties and potential applications in various medical diseases, including AD, PD, and stroke. Below are some potential applications of AuNPs in these neurological diseases: AuNPs may interact with Aβpeptides, preventing their aggregation into neurotoxic plaques, a hallmark of AD pathology. AuNPs may exert neuroprotective effects by scavenging ROS and reducing oxidative stress, which plays a role in the degeneration of dopaminergic neurons in PD. AuNPs may play a role in neuroprotection and regeneration after stroke. They modulate inflammatory responses, reduce oxidative stress, and promote neuronal survival and regeneration in the ischemic brain. AuNPs possess distinctive properties, rendering them a favorable choice for remedial interventions in such circumstances. However, several challenges must be addressed before their use becomes a widespread practice. Understanding AuNPs’ mechanisms in treating neurological disorders: The complete comprehension of the mechanisms through which AuNPs can be an effective treatment option for neurological diseases requires further research. Understanding these mechanisms is crucial for developing targeted and efficient treatments. Safety and efficacy: determining the safety and efficacy of AuNPs in AD, PD, and stroke treatment is essential.

Research on AuNPs in the context of neurological diseases holds significant relevance and timeliness due to several key factors. The increasing prevalence of these conditions underscores the urgency for innovative and effective therapeutic approaches. Current treatment options for many neurological diseases are limited, with existing drugs often providing only symptomatic relief and not addressing the underlying causes. This gap in effective treatments necessitates exploration of novel therapeutic strategies, such as those involving nanotechnology. Neuroinflammation is recognized as a common denominator in various neurological disorders. AuNPs, with their anti-inflammatory properties, offer a promising avenue for interventions that target this shared pathological feature [37,53]. This is particularly crucial in developing treatments for neurological diseases, where precise drug delivery is essential. Understanding and harnessing these properties could revolutionize treatment approaches for neurological disorders. AuNPs, being biocompatible and easily functionalized, are at the forefront of these advancements [30]. Research in this area aligns with the broader trends in leveraging nanotechnology for medical applications. Preclinical studies involving cellular and animal models have shown promise in demonstrating the efficacy of AuNPs in mitigating neurological damage and inflammation [53,89]. This preclinical evidence provides a strong rationale for advancing research into clinical applications. In summary, researching gold nanoparticles in the context of neurological diseases is relevant and timely due to the pressing health burden that is posed by these disorders, the inadequacy of current treatments, the commonality of neuroinflammation, challenges in drug delivery, and the unique properties of AuNPs [86,90].

### 3.1. Potential Use of AuNPs in the Treatment of AD

**Figure 2:**
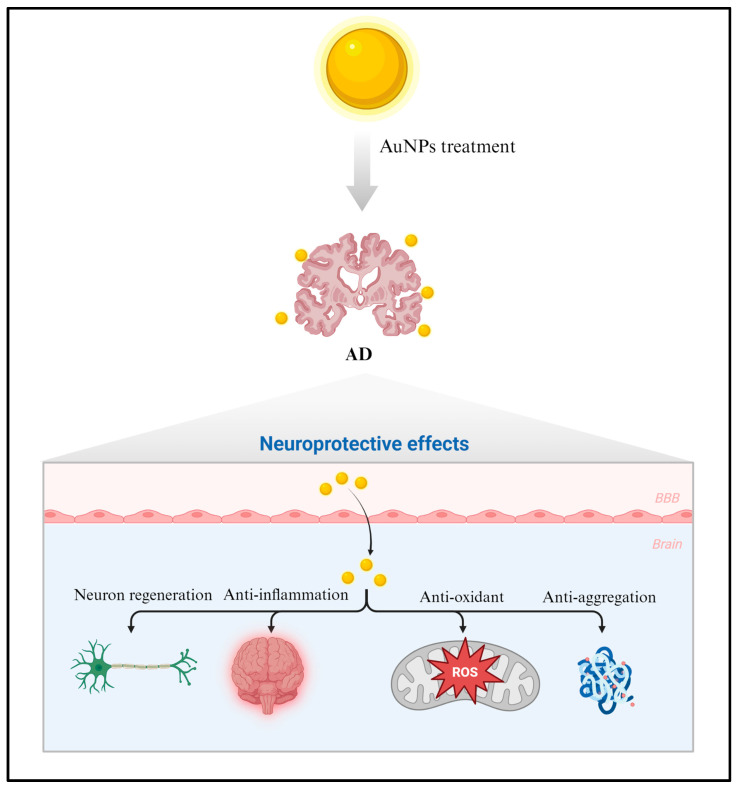
AuNPs exhibit neuroprotective characteristics and modulate inflammatory responses in AD. They can also regulate various cellular processes, including those related to neuroprotection. In AD, inflammation plays a pivotal role in neuronal damage. Hence, AuNPs can mitigate oxidative stress and inflammation, shielding neurons and maintaining cognitive faculties. Figure 2 created with BioRender.

AuNPs are being explored as a potential therapeutic strategy in the treatment of AD due to their unique properties [14,91,92]. Some ways in which AuNPs could be used are discussed in this section. Neuroprotection: AuNPs can have neuroprotective effects by reducing inflammation and promoting the survival of neurons. This could help preserve cognitive function and slow down disease progression. Promotion of neurogenesis: in some research, AuNPs have been shown to promote neurogenesis, which could aid in regenerating damaged neurons. Modulation of immune responses: AuNPs can modulate immune responses, potentially reducing neuroinflammation, a common AD feature. Antioxidant effects: Some AuNPs exhibit antioxidant properties, which can help mitigate oxidative stress. Oxidative stress plays a role in the progression of AD, contributing to neuronal damage. Anti-aggregation: AuNPs might interfere with the aggregation of Aβ proteins, preventing the formation of toxic plaques that contribute to neuronal damage and cognitive decline. Drug delivery: AuNPs can be functionalized to carry drugs across the BBB and target specific regions in the brain that are affected by AD. They could deliver drugs that inhibit the formation of Aβ plaques or tau tangles, the pathological hallmarks of the disease. Metal chelation: AuNPs might act as metal chelators, binding to metals like copper and zinc that are involved in amyloid aggregation. This could disrupt the formation of amyloid plaques. Overall, AuNPs have demonstrated potential in AD research due to their anti-inflammatory and neuroprotective properties (Figure 2). Several studies have investigated the therapeutic effectiveness of AuNPs in neurodegenerative conditions such as AD. This neuroprotective effect is crucial for impeding disease progression and preserving cognitive function. AuNPs possess anti-inflammatory properties that have the potential to alleviate nerve inflammation. By regulating the inflammatory response, AuNPs could aid in safeguarding brain cells and enhancing overall brain function in individuals with AD.

**Figure 3:**
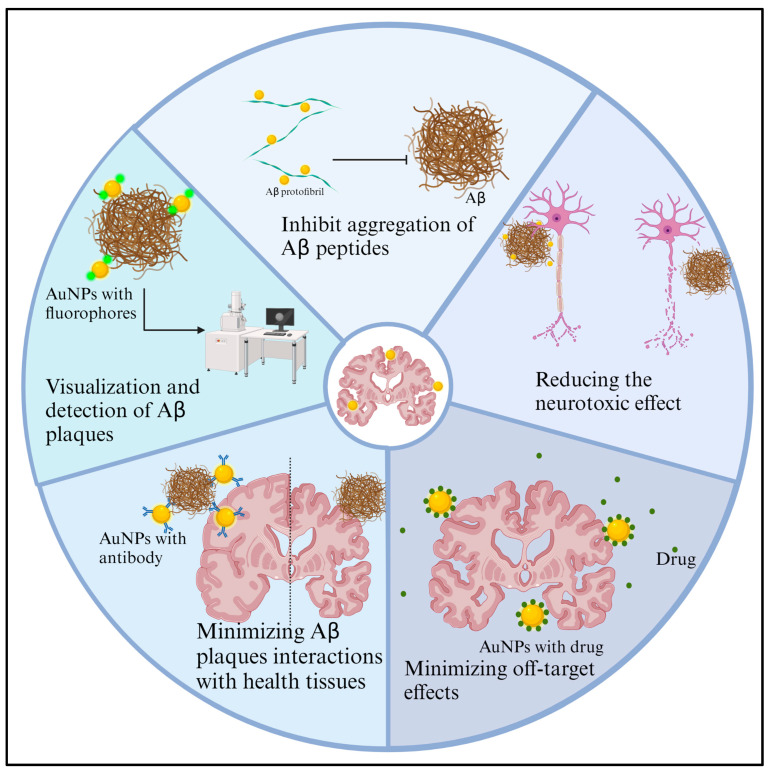
The research on AuNPs in AD suggests their potential as a targeted therapy. In vitro and preclinical in vivo studies reveal AuNPs’ ability to bind with Aβ aggregates, a key component of AD pathology. Functionalized AuNPs with specific ligands or peptides effectively interact with Aβ plaques, potentially inhibiting their aggregation. Figure 3 was created with BioRender.

In vitro and preclinical in vivo studies have suggested the binding of AuNPs with Aβ in the context of AD. These studies explore the potential of AuNPs as a targeted therapy for AD based on their interaction with Aβ (Figure 3). Several in vitro studies have demonstrated the ability of AuNPs to bind with Aβ aggregates. Functionalized AuNPs with specific ligands or peptides that have an affinity for Aβ have been shown to interact with and bind to Aβ plaques effectively [66]. Some studies suggest that AuNPs may inhibit the aggregation of Aβ peptides, a crucial step in forming the amyloid plaques that are characteristic of AD [93]. Preclinical in vivo studies, often conducted in transgenic mouse models of AD, have explored the targeting capabilities of AuNPs [92]. The interaction between AuNPs and Aβ has been explored for diagnostic imaging purposes. AuNPs that are functionalized with imaging agents, such as fluorophores or contrast agents, can enable the visualization and detection of Aβ plaques in vivo [94]. Functionalizing AuNPs with ligands or peptides with a high affinity for Aβ allows for targeted delivery [66,92]. This specificity is crucial for minimizing off-target effects and enhancing the therapeutic impact on Aβ pathology. By binding to Aβ, AuNPs may reduce the neurotoxic effects that are associated with Aβ aggregates [94]. This could potentially lead to a neuroprotective effect and mitigation of AD-related pathology. The basis for targeted therapy lies in the surface modifications of AuNPs. This functionalization allows AuNPs to target Aβ aggregates specifically. Together, these studies and reviews demonstrate ongoing research to elucidate further the mechanistic and therapeutic potential of AuNPs in addressing the Aβ-related pathology in AD. Yang et al. (2023) present a study that suggests that a designed hybrid nanomaterial, AuNPs@PEG@MIL-101, has promising characteristics for potential therapeutic applications in AD by targeting and modulating the aggregation of Aβ [95]. The nanomaterial exhibited the potential to decrease intracellular Aβ40 aggregation and reduce the amount of Aβ40 that is immobilized on the cell membrane. In cellular models using PC12 cells, AuNPs@PEG@MIL-101 demonstrated protective effects against Aβ40-induced microtubular defects and cell membrane damage. Lv et al. (2024) describe in their study an innovative electrochemical detection method utilizing a double amplification strategy involving gold nanoparticles (Au-MXene) as the electrode substrate and a covalent organic framework (COF)-based probe for enhanced sensitivity in detecting Aβ_1–42_ oligomers [96]. Warerkar et al. provide a comprehensive overview of the field, emphasizing the potential of coated nanoparticles for inhibiting amyloid fibril formation and addressing the challenges that are associated with drug development for neurodegenerative diseases [97]. Chang et al. (2024) present a novel approach for a biocompatible and stable conjugation of cysteine-Aβ peptides with AuNPs to detect femtomolar levels of Aβ peptides in human plasma. The ability to identify early-stage Aβ oligomerization and redirect the Aβ aggregation pathway holds therapeutic implications for AD. The findings are anticipated to contribute to revolutionary approaches in detecting and inhibiting Aβ aggregation at an early stage, potentially impacting the development of AD therapeutics [98].

The administration of AuNPs in a complex, such as a nanoparticle–drug conjugate or a multifunctional nanocomplex, holds the potential for greater therapeutic significance in various biomedical applications [32,99], including neurological disorders. Complexes can be designed to enhance the delivery, targeting, and therapeutic effects of AuNPs. Combination therapy: Forming complexes allows for the integration of multiple therapeutic components, such as drugs, imaging agents, or targeting ligands, into a single system. This approach enables combination therapy, where AuNPs are carriers for various therapeutic agents [100]. This could mean combining neuroprotective drugs and anti-inflammatory or imaging agents for neurological disorders to achieve synergistic effects. Targeted drug delivery: Incorporating targeting ligands on the surface of AuNP complexes enhances their specificity for particular cells or tissues [32], including those that are affected by neurological disorders. Targeted drug delivery can increase the therapeutic payload at the desired site, minimizing off-target effects and improving the efficacy of the treatment. For example, in AD, targeting Aβ plaques specifically with AuNP complexes could enhance drug delivery to affected areas [87]. Functionalization for specific interactions: The surface of AuNP complexes can be tailored with functional groups for specific interactions with biological targets or to enhance cellular uptake [101]. Functionalization allows for precise customization of the AuNP complex’s properties, influencing its interactions with biological systems [102]. This can be exploited to improve cellular uptake, target specific cell types, or modulate the release kinetics of therapeutic payloads. Reduced toxicity: Complexation can be designed to facilitate the potential toxicity that is associated with bare AuNPs [30]. Minimizing toxicity is essential for safe clinical applications. Complexes can include biocompatible coatings or materials to improve overall safety profiles. These studies highlight the potential of AuNPs in complex forms for AD treatment. Chiral properties, functionalization with specific compounds (such as mimosine), and conjugation with other therapeutic agents (like tetrahydroacridine derivatives) showcase the versatility and customization of AuNPs for therapeutic outcomes. Hou et al. (2020) conducted a study and highlight the promising therapeutic potential of chiral gold nanoparticles, particularly the D-enantiomer, in inhibiting Aβ_42_ aggregation and rescuing memory deficits in a mouse model of AD. These nanoparticles’ design, ability to cross the BBB, and biocompatibility make them a potential avenue for future AD therapeutics [92]. The study by Anand et al. (2021) indicates that mimosine-functionalized gold nanoparticles (Mimo-AuNPs) hold promise as a therapeutic intervention for AD. The inhibition of Aβ fibrillization, promotion of neuronal viability, reduction in tau protein phosphorylation, and reduction in oxyradicals suggest potential benefits in addressing the vital pathological features of AD. The ability of Mimo-AuNPs to cross the blood–brain barrier further enhances their potential as a targeted treatment strategy [93]. The study by Mojzych et al. (2023) explores the potential of a new tetrahydroacridine derivative (CHDA) and its conjugation with AuNPs for treating AD. The adsorption of CHDA onto gold surfaces and its conjugation with AuNPs contribute to its therapeutic potential for AD treatment through acetylcholinesterase inhibition [103]. Kim et al. (2023) describe a study exploring the neuroprotective effects of anthocyanin-loaded poly (ethylene glycol)-AuNPs in AD. Neuroprotective effects: Anthocyanin-loaded PEG-AuNPs (AnPEG-AuNPs) reduced Aβ_1–42_-induced neuro-inflammatory and neuroapoptosis markers. The neuroprotective effect was attributed to inhibiting the p-JNK/NF-κB/p-GSK3β signaling pathway. The results suggest that PEG-coated gold anthocyanin nanoparticles could be a new therapeutic agent in nanomedicine, particularly for preventing AD [83]. In summary, administering AuNPs in a complex enhances their therapeutic significance by allowing for combination therapy, targeted drug delivery, improved pharmacokinetics, specific interactions, multimodal imaging, and reduced toxicity.

These papers examine the use of AuNPs in treating AD. The following is a summary of the main content of Table 1: Dos Santos Tramontin et al. (2020) showed that utilizing AuNPs as therapeutics can reverse brain damage in an AD model [104]. Moreover, Zhou et al. (2022) pointed out that peptide-modified AuNPs can inhibit the fibril formation of Aβ and remove copper ions [105]. In 2023, a study revealed a new synthesized functionalized penetrating peptide–chondroitin sulfate AuNP, which could serve as an anti-AD drug. These particles are designed to penetrate the BBB [106]. Moreover, Hou et al. (2020) reported a study that pointed out that chiral AuNPs can selectively improve memory deficits in AD mouse models [92]. In 2021, Chiang et al. found that AuNPs have anti-inflammatory effects on oxidative stress that is caused by human neural stem cells being exposed to Aβ_1–42_ [53]. Sanati et al. (2019) reported that AuNPs impact Aβ-induced AD in a rat model involving the STIM protein [107]. These studies indicate that AuNPs may have potential therapeutic applications in AD and multiple mechanisms of action in the pathophysiology of AD.

### 3.2. Prospects for the Application of AuNPs in the Treatment of PD

The application of AuNPs in the treatment of PD holds several promising prospects, although further research and clinical studies are needed to demonstrate their effectiveness [38,39] entirely. Some potential avenues for their use in PD are discussed in this section. Neuroprotection: AuNPs with antioxidative properties could mitigate oxidative stress, a critical factor in PD’s degeneration of dopamine-producing neurons [108]. This neuroprotection could help slow disease progression. Neurogenesis promotion: some AuNPs have been associated with promoting neurogenesis, which could aid in regenerating dopamine-producing neurons that are lost in PD [109]. Inflammation modulation: AuNPs may help modulate neuroinflammation, which is increasingly recognized as a contributor to PD [15]. Reducing inflammation could potentially slow disease progression. Antioxidant effects: AuNPs with antioxidant properties can mitigate oxidative stress, a factor contributing to the death of dopamine-producing neurons in PD. Anti-aggregation: AuNPs, like AD, may hinder the aggregation of misfolded α-synuclein proteins, which is a characteristic of PD [110]. This could prevent the formation of toxic protein aggregates. Drug delivery: AuNPs can be engineered to carry neuroprotective drugs or disease-modifying agents to the brain, targeting specific areas that are affected by PD [111]. This targeted drug delivery could significantly enhance therapeutic efficacy and reduce potential side effects. Enhancing drug penetration: AuNPs can improve the penetration of drugs across the BBB, which is often a challenge in treating neurological disorders [86]. This could enable more effective delivery of existing PD medications.

**Figure 4:**
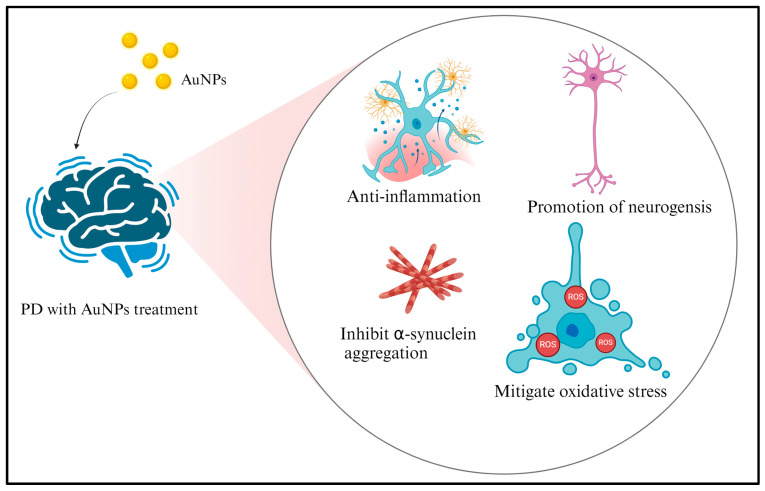
The neuroprotective and anti-inflammatory attributes of AuNPs are crucial for the development of treatments for PD. There are potential benefits of AuNPs for the treatment of neurodegenerative conditions like PD. Studies have shown that AuNPs can secure neurons from degeneration, which is essential for maintaining motor function and slowing the progression of PD. The preservation of neuronal integrity is a significant objective in the treatment of neurodegenerative ailments. Modulating the inflammatory response aids in preserving brain cells and optimal brain function, which is vital for individuals suffering from PD. Figure 4 created with BioRender.

AuNPs have been examined for their potential to protect neurons and reduce inflammation in neurodegenerative disorders, such as PD (Figure 4). Studies have demonstrated that AuNPs have neuroprotective properties, which are vital for maintaining motor function and delaying the progression of PD. Neuroinflammation, a crucial mechanism in the progression of neurodegenerative diseases like PD, can be alleviated by AuNP treatment. AuNPs possess anti-inflammatory properties, indicating their potential role in reducing brain inflammation. By regulating the inflammatory response, they can safeguard brain cells from harm and maintain optimal brain function.

Table 2 summarizes the use of AuNPs in PD models and their neuroprotective effects. Hu and collaborators have described the neuroprotective effect of AuNP composites in the PD model. Their research suggests that these composites have the potential for neuroprotection in PD [112]. Da Silva Corneo and colleagues (2020) explore the assessment of the effects of AuNPs on an animal model of PD through behavioral and oxidative parameter measurements. Their results show that AuNPs impact PD models, possibly affecting behavior and oxidative stress [113]. In 2019, a study indicated the application of gold nanoclusters in treating PD. This research investigated the therapeutic impact of gold nanoclusters on PD, and the findings indicated that gold nanoclusters hold promise for PD treatment [109]. Liu et al. (2020) demonstrated the use of active targeted AuNP composites in ameliorating cognitive and behavioral impairment in mice models of PD. This suggests that the composite can improve PD symptoms and positively impact behavior and cognitive function [114]. Studies have found that these biosynthetic AuNPs are neuroprotective against PD, showing promising results in both in vivo and in vitro models [15]. Overall, these studies show that AuNPs and their composites have potential application value in treating PD and may lead to the improvement of the symptoms and quality of life of patients with PD.

### 3.3. Potential of AuNPs for Neuroprotection after Stroke

**Figure 5:**
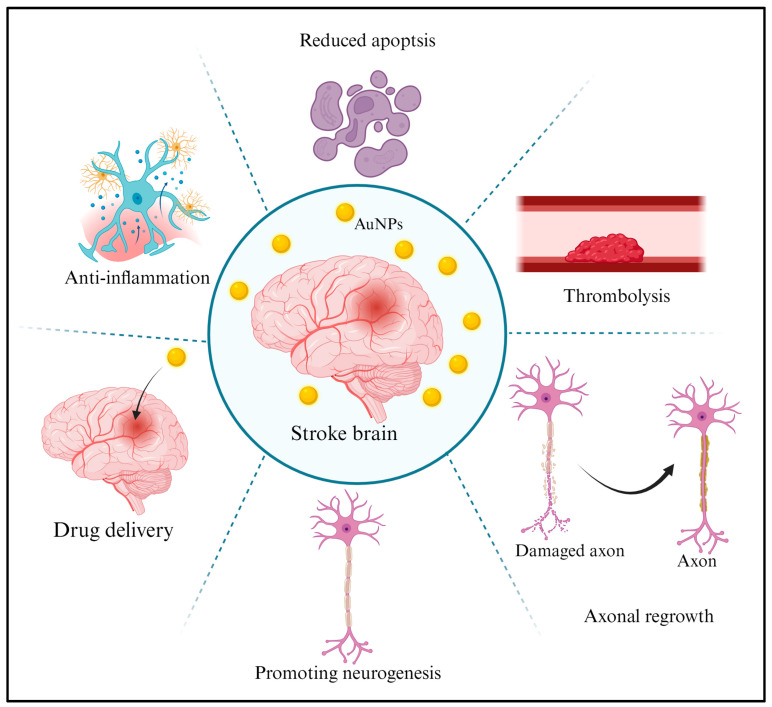
The neuroprotective and anti-inflammatory properties of AuNPs provide a promising avenue for treating stroke. Research in this field has immense potential for developing innovative therapies that can reduce brain damage, improve outcomes, and accelerate the recovery process for stroke patients. It is essential to continue studying and advancing the application of AuNPs and similar nanoparticles to explore their effectiveness in treating stroke and other neurological conditions further. Figure 5 created with BioRender.

AuNPs show potential for neuroprotection after a stroke due to their unique properties [115]. Some ways in which AuNPs could be used are discussed in this section. They could aid in brain tissue repair and functional recovery after stroke-induced damage. They could carry neuroprotective drugs that enhance neuronal survival and recovery or anti-inflammatory agents to reduce post-stroke inflammation. Reduced apoptosis: AuNPs could inhibit apoptosis in neurons, preserving cell viability and limiting damage. Axonal regrowth: AuNPs might promote the regrowth of damaged neuronal axons, which are crucial for restoring connectivity in the brain [76]. Thrombolysis: AuNPs could be used as carriers for thrombolytic agents to target and dissolve blood clots, which cause ischemic strokes [116]. Brain repair: AuNPs might enhance brain tissue regeneration and repair, promoting recovery from stroke-induced damage [115]. Consequently, the neuroprotective and anti-inflammatory effects of AuNPs hold potential for stroke treatment (Figure 5). AuNPs have been investigated for their capacity to preserve neurons from damage and death before and after stroke [115]. AuNPs protect the integrity of brain cells, thereby reducing the severity of brain damage during stroke. Inflammation is a significant contributor to secondary damage following a stroke, because the increased inflammation can exacerbate brain damage. AuNPs possess anti-inflammatory properties and can alleviate inflammation in stroke-damaged brain tissue [117]. Through the modulation of the inflammatory response, AuNPs show promise in shielding neurons and enhancing the overall recovery process following a stroke.

The research mentioned above discusses the applications and effects of AuNPs in stroke. The following is an overview of the main contents of Table 3: Zheng et al. (2019) showed that the inherent effect of AuNPs on injured rats’ cortical neurons, induced by oxygen–glucose deprivation/reperfusion, was studied. The study explored the effects of AuNPs on neuronal damage, potentially informing treatments for conditions such as stroke [118]. In 2020, Nazarian et al. reported the effect of mesenchymal stem cells that were coated with modafinil on neurological defects in rats after middle cerebral artery occlusion was studied. The study explored the use of AuNPs in stroke treatment and pointed to their positive impact on neurological recovery [119]. Huang et al. (2022) proposed developing a drug delivery system to treat cerebral ischemia–reperfusion injury using a gold nanocarrier wrapped with dendrimers that were modified with gastrodin. The study explored the potential therapeutic effects of this novel drug-delivery system on brain injuries [120]. Rathore et al. (2020) demonstrated that collagen nanoparticle-mediated brain delivery of silymarin was explored to treat cerebral ischemia–reperfusion injury. The study discussed the effectiveness of this drug delivery system in mitigating brain damage, potentially providing new avenues for neuroprotection after stroke [121]. In general, these studies explored the application of AuNPs in stroke and proposed different methods and mechanisms, which are expected to provide new ideas for treating neuroprotection-related diseases.

The potential effectiveness of AuNPs in neurological diseases may vary depending on the specific characteristics and mechanisms that are involved in each disease. AuNPs in neurological applications are still in the preclinical stages, and the optimal stage of the disease for AuNP intervention may not be conclusively determined. AuNPs that are designed to target Aβ plaques might be more effective in the early stages of AD when Aβ accumulation begins [122]. Early intervention could potentially prevent or slow down the progression of Aβ aggregation and the associated neurodegeneration. In PD, where there is a loss of dopaminergic neurons, AuNPs with neuroprotective properties might be beneficial [112,114]. Early intervention, primarily when the initial symptoms manifest, could potentially protect neurons and slow down the progression of the disease. In ischemic stroke, AuNPs may be administered in the acute phase to mitigate neuronal damage and promote neuroregeneration [117,123]. The immediate aftermath of a stroke presents a critical window for interventions to limit the extent of injury. For chronic neurodegenerative diseases, such as AD or PD, the continuous administration of AuNPs with neuroprotective and anti-inflammatory properties may be considered to provide ongoing support and potentially slow disease progression [66]. It is important to note that the effectiveness of AuNPs can be influenced by various factors, including the size, surface modifications, and functionalization of the nanoparticles [29], as well as the specific molecular and cellular mechanisms underlying each neurological disorder. The stage at which AuNPs can be most effective may vary based on the intended therapeutic strategy—whether it is focused on preventing disease onset, slowing progression, or providing symptomatic relief.

## 4. Cellular and Animal Research

### 4.1. Experimental Results in Cellular Models

Using AuNPs for neuroprotection in cellular models of various neurological conditions has yielded promising experimental results in many research studies [67,90,91,124]. Some key findings include the following: Anti-inflammatory effects: AuNPs have been shown to suppress the production of pro-inflammatory cytokines (e.g., TNF-α, IL-6) in cellular models of neuroinflammation [125]. This suggests their potential to modulate immune responses and reduce inflammation-associated damage. Oxidative stress reduction: Certain AuNPs possess antioxidant properties and can reduce oxidative stress markers in neuronal cells [90,126,127]. This is significant in protecting cells from oxidative damage, which is implicated in neurodegenerative diseases. Cell viability improvement: In cellular stress or toxicity models, AuNPs have demonstrated the ability to enhance cell viability and prevent cell death [68]. This indicates their potential to maintain neuronal health and survival. Neurite outgrowth: AuNPs have been observed to promote neurite outgrowth in neuron-like cells [128]. This suggests their role in supporting neuronal connectivity and regeneration. β aggregation inhibition: In AD models, certain AuNPs have exhibited the ability to interfere with the aggregation of amyloid beta proteins, which form toxic plaques [53,93]. This could potentially slow disease progression. Neurogenesis stimulation: AuNPs have shown the potential to stimulate neurogenesis in neural stem cells, contributing to generating new neurons [129]. Mitochondrial protection: AuNPs have been found to protect mitochondria from damage that is induced by stressors, indicating their role in maintaining cellular energy production and function [55,91]. Modulation of signaling pathways: AuNPs have demonstrated an ability to affect signaling pathways related to neuroprotection, cell survival, and inflammation [14]. Selective uptake: cellular uptake studies reveal that cells can take up certain types of AuNPs, making them suitable for targeted interventions [130]. It is important to emphasize that while these experimental results are promising, they primarily come from in vitro cellular models, which do not fully replicate the complexity of living organisms. Further research is needed to validate these findings in more physiologically relevant models and to determine the potential translation of AuNP-based interventions to clinical settings.

### 4.2. Experimental Results in Animal Models

Research exploring the use of AuNPs for neuroprotection in animal models of neurological conditions has provided valuable insights [67,114]. Below are some critical experimental findings: Stroke models: In animal stroke models, AuNPs have demonstrated neuroprotective effects [131]. They have been shown to reduce the infarct size, improve neurological deficits, and promote functional recovery. AuNPs’ anti-inflammatory and antioxidant properties contribute to these positive outcomes. Neuroinflammation modulation: AuNPs have been found to suppress neuroinflammation in animal models of neurodegenerative diseases [14,66]. They can reduce the levels of pro-inflammatory cytokines and microglial activation, indicating potential anti-inflammatory benefits. Cognitive enhancement: In animal models of cognitive decline, AuNPs have shown the potential to improve memory and cognitive function [129]. This effect is attributed to their ability to mitigate oxidative stress and inflammation. PD models: Some studies suggest that AuNPs could protect dopaminergic neurons in animal PD models [114]. They have shown the potential to restore dopamine levels and motor function, likely through their antioxidative and neuroprotective properties. AD models: AuNPs have exhibited promising results in animal models of AD. They have been shown to reduce Aβ plaque deposition, improve cognitive deficits, and enhance memory [92]. Nerve regeneration: Animal nerve injury models have indicated that AuNPs could support nerve regeneration and functional recovery. They might aid in remyelination and axonal regrowth. Biocompatibility and safety: Many studies have assessed the biocompatibility and safety of AuNPs in animal models. Their results show that properly engineered AuNPs, in appropriate doses, can have minimal toxic effects. BBB permeability: AuNPs have demonstrated the ability to cross the BBB in animal models, enabling them to directly reach the brain and exert their neuroprotective effects [33,132]. It is important to note that while these animal model results are promising, the translation of findings to human clinical applications is complex.

**Figure 6:**
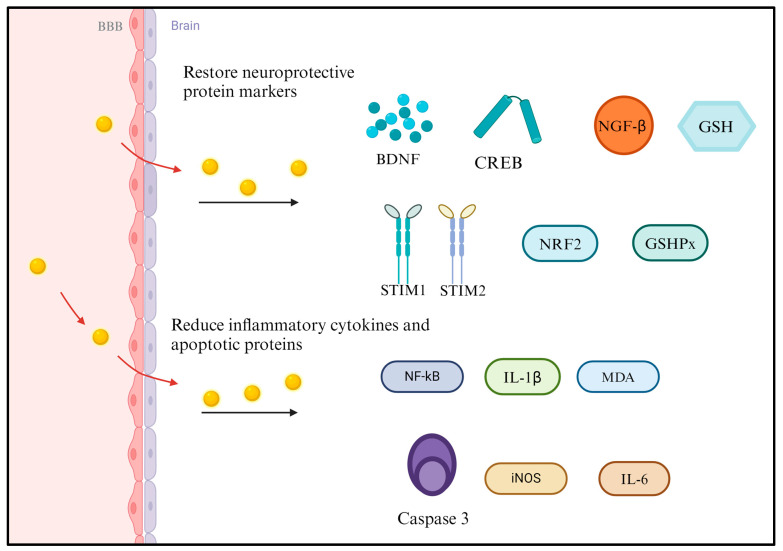
The signal transduction pathways involved in AuNP-mediated neuroprotection mechanisms. This includes inhibiting the formation of pathological proteins, anti-inflammatory effects, and potential therapeutic effects. These pathways provide valuable insights into innovative therapeutic strategies of AuNPs in neurology. Figure 6 created with BioRender.

Based on the above content, these studies demonstrate the diverse applications of AuNPs in neuroprotective mechanisms, providing potential therapeutic strategies in neurological diseases such as AD, PD, and stroke (Figure 6). However, based on general knowledge of nanoparticle interactions with biological systems, including cells in the nervous system, we can discuss potential signal transduction pathways that might be implicated. Anti-inflammatory signaling: AuNPs have been reported to exhibit anti-inflammatory effects in the context of oxidative stress that is caused by Aβ in AD. The anti-inflammatory response could involve pathways such as NF-κB or MAPK, which are commonly associated with inflammatory responses. Antiapoptotic signaling: The neuroprotective effects observed in studies might be linked to antiapoptotic signaling pathways. Reducing neuronal damage could involve the PI3K/Akt pathway, which promotes cell survival and inhibits apoptosis. Neuroprotective effects observed in PD models may involve neurotrophic signaling pathways, such as the BDNF/TrkB pathway, known for promoting neuronal survival and growth. Oxidative stress response: the neuroprotective effects observed in stroke models could be associated with pathways responding to oxidative stress, such as the Nrf2/ARE pathway, which regulates antioxidant defenses. Cellular uptake and intracellular signaling: as reported in some studies, the internalization of AuNPs into cells might activate intracellular signaling cascades, potentially involving endocytic pathways and downstream signaling events. It is crucial to note that the specific pathways that are involved can vary based on the size, shape, surface functionalization, and other properties of AuNPs, as well as the cell type and disease context. Nanomedicine is dynamic, and ongoing research is necessary to elucidate the intricate molecular mechanisms underlying AuNP-mediated neuroprotection.

## 5. Conclusions

This review explores the potential of AuNPs as promising therapeutic candidates for neuroprotection and anti-neuroinflammation strategies in neurological disorders. The article emphasizes the importance of tackling neuroinflammation, toxicity, and neuroprotection in these conditions. It also explores the distinctive features of AuNPs that render them well suited for biomedical usage. This review underscores the need for innovative approaches to targeting neuroinflammation, given its involvement in various neurological disorders. It introduces AuNPs as multifunctional agents that are capable of addressing neuro-inflammatory responses through various mechanisms. The discussion revolves around how AuNPs can modulate signaling pathways, mitigate oxidative stress, inhibit pro-inflammatory cytokines, and promote anti-inflammatory factors, ultimately contributing to neuroprotection. The unique physicochemical properties of AuNPs, including their size, surface chemistry, and biocompatibility, are highlighted as critical factors that make them versatile for designing targeted therapeutic interventions. Methods of synthesizing and functionalizing AuNPs are explored, emphasizing their impact on biocompatibility, stability, and specific interactions with neural cells. The review delves into the mechanisms through which AuNPs exhibit neuroprotective effects. These mechanisms include antioxidant properties, anti-inflammatory effects, mitochondrial protection, antiapoptotic effects, neurotrophic effects, enhanced neuronal connectivity, modulation of signaling pathways, and potential reduction in protein aggregation. Collectively, these findings contribute to understanding how AuNPs can combat neuronal damage and promote cell survival. The safety profile of AuNPs is discussed, emphasizing their biocompatibility and limited cellular uptake at appropriate concentrations. Strategies to mitigate potential adverse effects, such as size optimization, surface functionalization, dosage control, and targeted delivery, are highlighted. The importance of comprehensive studies to assess long-term effects, biodistribution, and patient variability is underscored. This review presents the translational potential of AuNPs in clinical settings. Their ability to enhance drug delivery, cross the BBB, and improve therapeutic efficacy is explored. The multifunctionality of AuNPs for imaging and diagnostics is also discussed, highlighting their potential for personalized medicine. The importance of clinical trials, safety studies, and personalized approaches is highlighted, as the field advances toward harnessing the full potential of AuNPs for neuroprotection and anti-neuroinflammation strategies in neurological disorders. In conclusion, the potential of AuNPs to revolutionize neuroprotection approaches is groundbreaking. Their versatility, precision, and innovative applications promise to reshape how we approach neurodegenerative disorders and neurological injuries, ushering in a new era of targeted, effective, and personalized treatments that hold the potential to change lives and reshape the landscape of neurological care.

AuNPs in neurology are rapidly evolving, and several challenges and opportunities exist. Future research directions should address these challenges to advance the application of AuNPs in neurology. Tailoring AuNPs for specific disorders is challenging but essential. Investigating disease-specific molecular targets and designing AuNPs with surface modifications that enhance their specificity for particular neurological conditions are also important future directions, as is investigating the factors influencing their pharmacokinetics, including their size, surface modifications, and administration routes [32]. Developing AuNPs that are capable of multimodal interventions, combining therapeutic and diagnostic functions, poses challenges in design and optimization. Other research directions include the following: Explore the integration of therapeutic agents, imaging agents, and other functionalities regarding AuNPs to create versatile platforms for combined therapies. Conduct comprehensive, long-term toxicity studies in animal models, focusing on potential accumulative effects, immunogenic responses, and impacts on organ systems beyond the nervous system [133]. Fine-tune drug delivery aspects, such as the payload capacity, release kinetics, and targeted delivery, which remain a complex challenge. Investigate novel drug-loading techniques, develop controlled release systems, and enhance targeting strategies to optimize the therapeutic potential of AuNPs in drug delivery [32]. Utilize advanced techniques such as proteomics and systems biology to unravel the complex interactions between AuNPs and biological components, facilitating a more nuanced understanding of their behavior in biological environments [134]. Addressing these challenges and exploring these research directions will contribute to the responsible advancement of AuNPs in neurology, potentially unlocking innovative and effective therapeutic strategies for neurological disorders.

Overall, this comprehensive review underscores the burgeoning potential of AuNPs in revolutionizing neurological interventions. The amalgamation of innovative surface modifications, multifunctional capabilities, and targeted drug delivery strategies positions AuNPs as promising candidates for the next frontier in neurology. The elucidation of intricate molecular mechanisms, as discussed herein, not only enhances our understanding of AuNPs’ neuroprotective effects but also lays a robust foundation for future therapeutic endeavors. The reviewed literature indicates that AuNPs possess unique properties that make them versatile tools for effective treatments of neurological diseases. From mitigating neuroinflammation and modulating apoptosis to facilitating targeted drug delivery and imaging, AuNPs exhibit a spectrum of actions that can be tailored for specific neurological contexts [32,53,68]. The hypothetical case studies presented exemplify the potential of AuNPs to address the complexities of AD, PD, and stroke. Standing at the intersection of innovation and application, the findings presented in this review illuminate a pathway toward a paradigm shift in neurological treatments. The dynamic nature of AuNPs, their adaptability to multifunctional roles, and the potential for personalized interventions position them as agents of transformative change. Moving forward, the research community must continue to unravel the intricacies of AuNPs, bridging the gap between the bench and bedside to bring about a new era of neurology.

## Figures and Tables

**Table 1:** AuNPs demonstrate neuroprotective and anti-inflammatory qualities in AD.

Biological Model	Pathways	Targets/Mechanisms	References
AD model induced by 100 μg okadaic acid in male Wistar rats and then treated with 20 nm AuNPs at a dose of 2.5 mg/kg every 48 h for 21 days.	AuNPs prevented neuroinflammation, preserved mitochondrial function, restored antioxidant status, and improved cognitive impairment.	AuNP treatment restored abnormal tau phosphorylation, BDNF, NGF-β, IL-1β, ATP synthase activity, SOD, catalase activities, and glutathione (GSH) levels and maintained them at normal levels.	[104]
The effect of AuNPs on Aβ-induced cytotoxicity in human neuroblastoma SH-SY5Y cells.	AuNPs have the potential to serve as effective inhibitors of Aβ fibrillogenesis.	AuNPs showed a significant protective effect, suggesting that inhibiting Aβ fibrillation and copper ion chelation had a beneficial effect on neurons.	[105]
AuNP applications as an anti-AD drug in Aβ_1–40_-induced apoptosis in SH-SY5Y cells.	Inhibition of Aβ_1–40_ accumulation, reduction in Aβ_1–40_-induced apoptosis, protection against oxidative stress and cholinergic injury, inhibition of aberrant tau phosphorylation, and suppression of inflammatory response.	AuNPs reduced abnormal tau phosphorylation, inflammatory factors, and oxidative stress damage by regulating GSK3β, NF-κB signaling pathway, malondialdehyde (MDA), and glutathione peroxidase (GSH-Px) levels.	[106]
Inhibition effect of AuNPs (110 nM) on Aβ42 (40 μM)-induced neuronal death in SH-SY5Y cells. Effect of AuNPs (25 mg kg^−1^) on rescuing memory impairments in APP/PS1 mice.	AuNPs demonstrated the ability to protect neuronal cells from Aβ42-induced death in vitro and improve memory impairments in a mouse model of AD.	By inhibiting Aβ42 fibrillation and effectively crossing the BBB, these nanoparticles could offer a way to treat behavioral impairments.	[92]
The investigation concerns the potential therapeutic advantages of 10 ppm of AuNPs in a 3D cell culture model, utilizing human neural stem cells exposed to 5 μM of Aβ_1–42_.	The research shows that AuNPs effectively reduce inflammation and oxidative stress in hNSCs exposed to Aβ, specifically under 3D scaffold conditions.	The AuNPs led to the normalization of the expression of inflammatory cytokines (specifically TNF-α and IL-1β), NF-κB (p65), nuclear factor erythroid 2-related factor 2 (Nrf2), and aggregates in Aβ-treated human neural stem cells.	[53]
The possible therapeutic benefit of AuNPs in mitigating cognitive and memory deficits in a rat model induced by the Aβ model.	In the Morris water maze, rats who received treatments of Aβ and AuNPs demonstrated extended presence in the target quadrant, thus indicating memory retention enhancements.	The levels of critical proteins necessary for the survival and adaptability of neurons, including BDNF, CREB, and stromal interaction molecules (STIM1 and STIM2), were increased in rats subjected to Aβ and AuNP treatment.	[107]

**Table 2:** AuNPs have been found to possess neuroprotective and anti-inflammatory effects in PD.

Biological Model	Pathways	Targets/Mechanisms	References
The therapeutic effectiveness of the composites of AuNPs has been demonstrated in both in vitro (in PC12 cell cultures) and in vivo (in living organisms) models of PD.	In a model of PD, AuNP composites show neuroprotective effects.	The AuNPs are transfected into cells via endocytosis. This process inhibits apoptosis in PC12 cells and dopaminergic neurons, potentially preserving these cells from degeneration.	[112]
AuNPs were administered at 2.5 mg/kg (20 nm) for five consecutive days in 0.25 mg/kg reserpine-induced male C57BL/6 mice.	AuNPs have positive effects in reversing behavioral and oxidative stress parameters in a reserpine-induced PD model.	AuNPs reversed the behavioral and oxidative stress parameters observed in the reserpine-induced PD mice. Additionally, AuNPs partially improved neurotrophic factors that are crucial for neuronal survival.	[113]
AuNPs exhibit effective neuroprotective properties in cellular PD models and mouse PD models.	A new direction for the application of AuNPs in medicinal contexts, specifically in treating neurodegenerative disorders like PD.	AuNPs can potentially prevent α-synuclein fibrillation, provide neuroprotection in cell models, improve behavioral symptoms, and reverse dopaminergic neuron loss in a mouse model of PD.	[109]
AuNPs demonstrated substantial neuroprotective effects on both motor and non-motor aspects of the PD mouse model induced by MPTP (30 mg/kg intraperitoneal twice a week).	AuNPs showed a considerable neuroprotective impact to enhance the behavioral and cognitive deficits of mice with PD.	The treatment with AuNPs led to a reduction in the aggregation of α-synuclein in the substantia nigra. AuNP-treated mice exhibited improved long-term potentiation and exploration ability, positively impacting their cognitive and behavioral function.	[114]
In vitro, assays demonstrated the capacity of AuNPs to suppress inflammation in murine microglial BV2 cells. In vivo, studies established their beneficial effects in preventing neuroinflammation and improving motor coordination in PD-induced mice.	These findings suggest the potential therapeutic use of these AuNPs in treating PD, highlighting their neuroprotective and anti-inflammation properties.	The neuroprotective effects were assessed by measuring nitric oxide, prostaglandin E2 assays, and inflammatory cytokines (IL-6 and IL-1β). The results suggest that the AuNPs can mitigate inflammatory conditions induced by LPS in BV2 cells—moreover, gold nanoparticles alleviate neuroinflammation and improve motor coordination in the C57BL/6 mice induced with PD.	[15]

**Table 3:** AuNPs have been shown to possess neuroprotective and anti-inflammatory properties in cases of stroke.

Biological Model	Effects	Targets/Mechanisms	References
The impact of 20 nm AuNPs on neuronal injury and survival in primary rat cortical neurons during oxygen–glucose deprivation/reperfusion (OGD/R) injury.	AuNPs might exhibit neuroprotective effects and anti-inflammatory properties in OGD/R injury in rat cortical neurons.	AuNPs might modulate neuronal cell viability, cell survival, antiapoptotic pathways, mitochondrial oxygen consumption, autophagic processes, and neurotransmitter release during OGD/R injury.	[118]
Neuroprotective effects of 50 nm AuNPs (100 mg/kg/day) in a rat model of middle cerebral artery occlusion (MCAO).	AuNPs could enhance neuronal survival and neurotrophic factor levels in the MCAO model.	AuNPs significantly reduced brain infarct volume and apoptosis while increasing BDNF, GDNF, and NeuN levels in ischemic brain injuries.	[119]
Neuroprotective effects of AuNPs in the MCAO rats.	AuNPs notably reduced MCAO-induced apoptosis.	Pathological alterations in brain tissue and significant organs were identified through staining with hematoxylin and eosin. Apoptotic rat astrocytes and hypothalamic neurons were discovered using TUNEL staining and flow cytometry. AuNPs exerted anti-inflammatory and antiapoptotic effects against cerebral ischemia–reperfusion injury.	[120]
Nanoparticles enhanced their effectiveness in treating rat brain injury induced by cerebral ischemia and reperfusion.	The use of nanoparticles has resulted in improved drug bioavailability and targeted drug delivery, leading to enhanced neuroprotective effects.	Nanoparticles showed a significant reduction in the expression of inflammatory proteins NF-kB, iNOS, and apoptotic protein caspase-3 in the MCAO group.	[121]

## Data Availability

Not applicable.
